# Selective medial soft tissue release combined with tibial reduction osteotomy in total knee arthroplasty

**DOI:** 10.1186/s13018-017-0681-1

**Published:** 2017-11-14

**Authors:** Qian Tang, Hua-chen Yu, Ping Shang, Shang-kun Tang, Hua-zi Xu, Hai-xiao Liu, Yu Zhang

**Affiliations:** 1 Department of Orthopaedic Surgery, The Second Affiliated Hospital and Yuying Children’s Hospital of Wenzhou Medical University, 109, Xueyuanxi road, 325027 Wenzhou, China; 2 Department of Rehabilitation, The Second Affiliated Hospital and Yuying Children’s Hospital of Wenzhou Medical University, 109, Xueyuanxi road, 325027 Wenzhou, China; 3 Department of Clinical Medicine, Second Clinical Medical College, Wenzhou Medical University, 325000 Wenzhou, China

**Keywords:** Total knee arthroplasty, Medial collateral ligament, Tibial reduction osteotomy, Varus knee, Soft tissue release, Orthopedic surgery

## Abstract

**Background:**

To obtain the correct coronal alignment and balancing in flexion and extension, we established a selective medial release technique and investigated the effectiveness and safety of the technique during primary total knee arthroplasty (TKA).

**Methods:**

Four hundred sixty-six primary TKAs with varus deformity were prospectively evaluated between June 2013 and June 2015. A knee joint position similar to Patrick’s sign was used to release the medial structure. The medial release technique consisted of release of the capsule and the deep medial collateral ligament (dMCL) (step1), selective release of superficial medial collateral ligament (sMCL) or posterior oblique ligament (POL) (step 2), and selective tibial reduction osteotomy (step 3). Improvement of medial joint gap at each step and other clinical outcomes were evaluated.

**Results:**

Among the 466 knees, symmetrical gaps could be achieved by the limited release of the capsule and the dMCcL in 276 (59%) knees. One hundred fifty-two (33%) required additional sMCL release with 2–5 cm from the joint line distally or POL release. Thirty-eight (8%) necessitated an additional tibial reduction osteotomy. Anterior-medial release and 4-mm medial osteotomy contributed to more improvement of medial gap in flexion than in extension (each *p* < 0.01). Posteromedial release and posteromedial osteotomy contributed to more improvement in extension than in flexion (each *p* < 0.01). No specific complication related to our technique was identified.

**Conclusion:**

The technique of the tibial reduction osteotomy combined with medial soft structure release using Patrick’s sign is effective, safe, and minimally invasive to obtain balanced mediolateral and extension-flexion gaps in primary TKA.

## Background

Varus deformity is a common problem in total knee arthroplasty (TKA), and an uncorrected deformity has a bad influence on the longevity of the implants [1–4]. To obtain the correct coronal alignment and balancing in flexion and extension, a soft tissue release of the medial structures is frequently needed in severe varus knee [5]. There is a consensus among authors that a medial release should be performed sequentially depending on the degree of varus deformity [6–9].

There exists a controversy in the methods and the order of soft tissue release to achieve balanced gap during TKA of varus deformed osteoarthritic knee [9, 10]. Most surgeons suggested deep layer of the medial collateral ligament (dMCL) release from the proximal tibial attachment as their first step of medial soft tissue release in varus knees. At the next step, various and complex protocols of medial release have been reported including release of superficial layer of the medial collateral ligament (sMCL), posterior oblique ligament (POL), posteromedial capsule, semimembranosus (SM), and pes anserinus, as well as tibial reduction osteotomy [11, 12].

In knees with severe medial tightness, the possibility of complete detachment of the sMCL by an extensive subperiosteal release during primary TKA has been a concern [13]. Extensive release techniques can lead to instability and also seem to be less effective. The sMCL is the primary restraint to the valgus force of the medial side of the knee [11, 12, 14]. Conservation of the distal attachment of sMCL is considered to be critical to maintain the joint stability when possible. Reduction osteotomy is known as a soft tissue-sparing technique for achieving soft tissue balancing [15], and it is capable of reducing the amount of release required to balance the knee, minimizing the risk of medial over release.

As minimal and efficacious release is the prerequisite of the ideal soft tissue-balancing technique in TKA, we describe steps to release the medial elements mini-invasively by the method of the medial proximal tibial reduction osteotomy combined with soft tissue release during TKA in severely deformed knees. It consists of dMCL release, selective release of sMCL or POL, and selective tibial reduction osteotomy.

In addition, release of posteromedial corner structures, such as POL and SM tendon, are often cumbersome in severe varus knees [15]. In this study, we sought to (1) introduce a knee joint position similar to Patrick’s sign in favor of sufficient exposure of the medial structure, (2) determine whether the technique of tibial reduction osteotomy combined with medial soft structure release is effective and minimally invasive, and (3) provide a fine control of mediolateral balance as well as extension-flexion balance by selective soft structure release and selective tibial reduction osteotomy.

## Methods

Approval to conduct the study was gained from the local Human Research Ethics Committee. All patients involved in our study signed the informed consent.

The data of 504 consecutive varus knees of 436 patients who underwent primary TKAs was prospectively collected between June 2013 and June 2015. Knees of neutral alignment without any soft tissue release, valgus knees, extraarticular varus deformity, and primary TKAs with severe preoperative ligament instability that needed constrained TKAs were excluded. The cases with large defects after tibial resections were also excluded. The large defects, as defined by Dorr, may occupy 25% or more of the component undersurface and involve a deficit deeper than 5 mm [16].

Among them, 38 knees of 34 patients were lost to follow-up. Thus, 466 knees of 402 patients (92%) comprised the cohort of this study with a mean follow-up of 1.3 years (range, 1–2 years). The mean age of the patients was 63.7 years (range, 55–76 years). All patients diagnosed with knee osteoarthritis were included.

All the TKAs were performed by a senior author in our institute using a medial parapatellar approach. The bone resections were performed before any soft tissue release procedures. Both cruciate ligaments were removed for a posterior cruciate-substituting system (Smith & Nephew, Genesis II). Femoral distal resection was made at 5°–7° valgus by an intramedullary alignment guide with respect to the femoral anatomic axis. The femoral external rotation was decided using the transepicondylar axis and anteroposterior axis. A tibial cut was made perpendicularly to the mechanical axis of the tibia, approximately 9–11 mm in thickness from the lateral tibial cortex by extramedullary guided manner. If there were bone defects that require tibial resections of more than the planned, another 2-mm resection of the tibia was conducted with the joint line being slightly lower (1 mm).

**Fig. 1:**
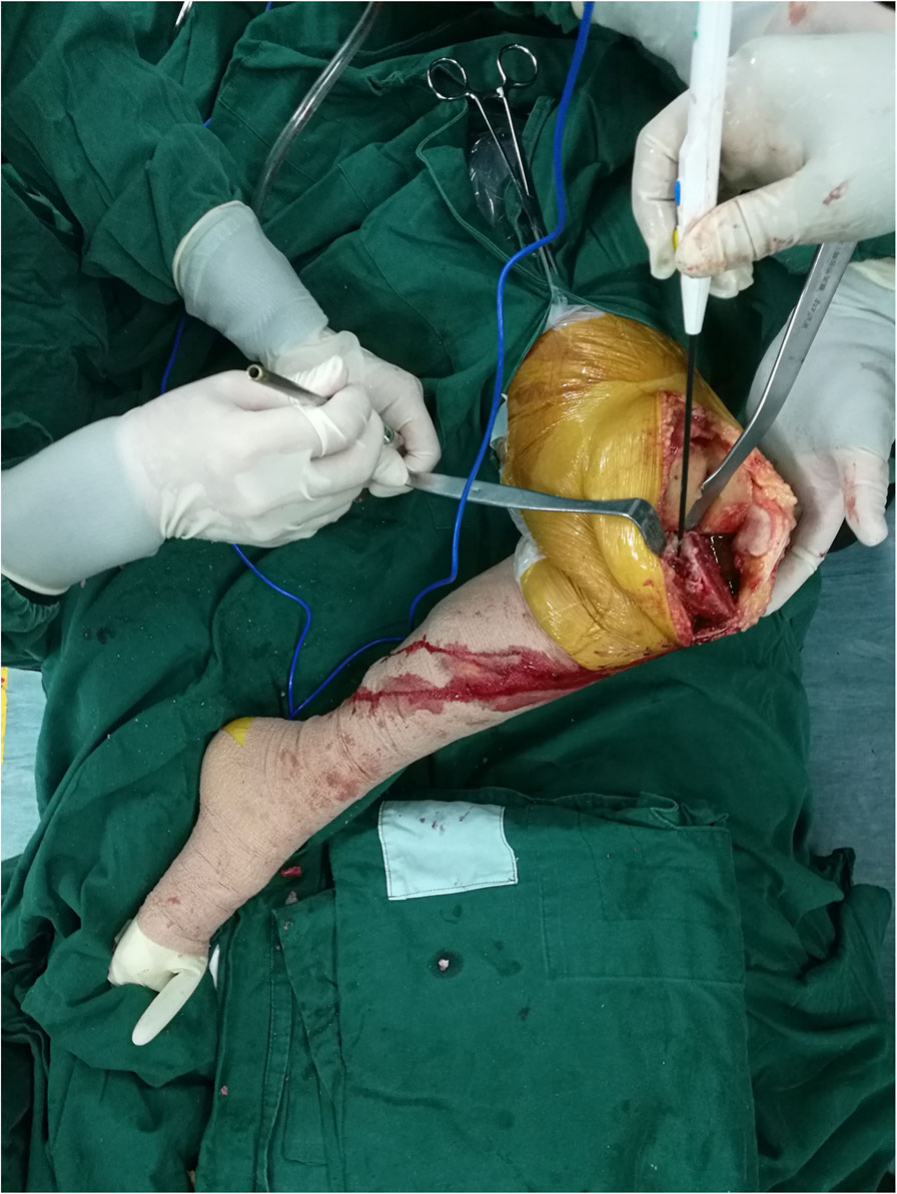
A knee joint position similar to Patrick’s sign was used by pushing the ankle joint to the medial side and the knee joint to the lateral side, which was in favor of exposing the medial structure of the knee

**Fig. 2:**
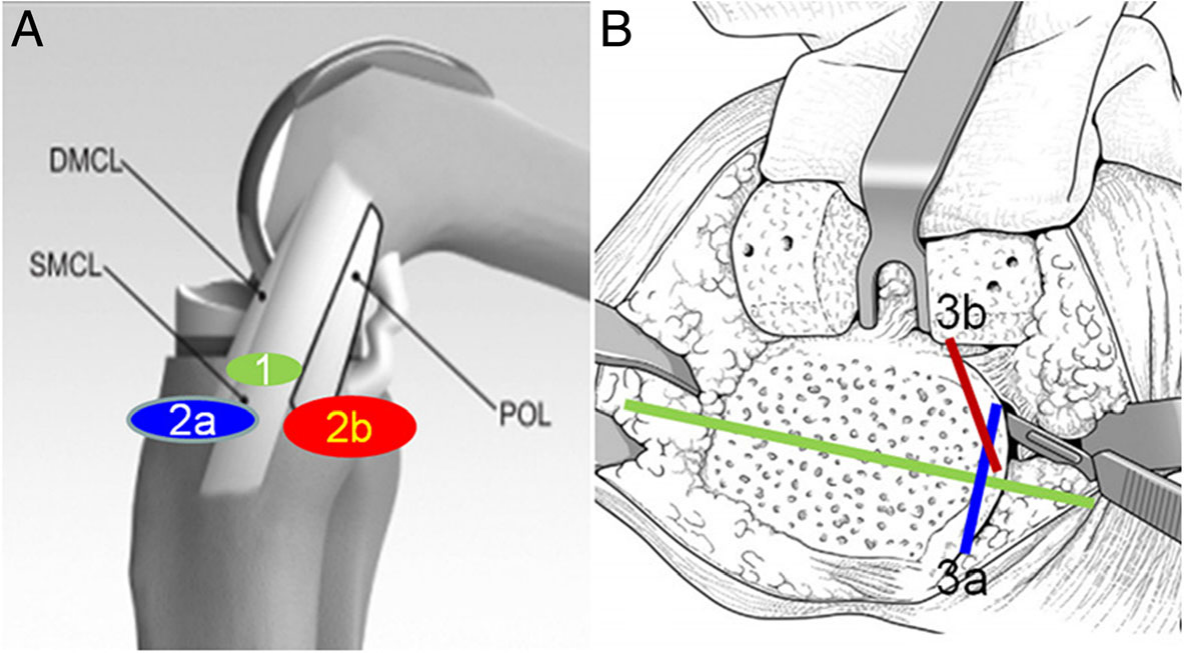
A diagram illustrates the step by step procedures of medial structure release. **a** The release of medial soft tissue structures which are consisted of dMCL release (Step 1), selective release of anterior-medial (Step 2a) or posteromedial structure (Step 2b); **a** The selective reduction osteotomy which consisted of selective medial (Step 3a) or posteromedial tibia reduction osteotomy (Step 3b)

A knee joint position similar to Patrick’s sign was used to release the medial structure (Fig. 1), which is performed by having the knee flexed and the thigh abducted and externally rotated. In this position, the surgical procedures for releasing medial structure could be easily performed with pulling away the soft tissue by a retractor. The peripheral osteophytes of the distal femur and proximal tibia were removed. The dMCL was released at the menisco-capsular junction, and the medial capsule was regionally released from the edge of the tibial joint surface, which was not beyond 2 cm from the joint line distally (step 1). If the medial gap is still tight, two different methods were further selected according to the medial tightness of flexion and extension gaps: release of anterior-medial or posteromedial structure. Accordingly, medial tightness in flexion only was addressed by releasing the anterior-medial portion of the capsule, anterior arm of SM tendon, and proximal division of the sMCL, while medial tightness in extension only was addressed by releasing the posteromedial structure including the posteromedial capsule, POL, anterior arm, and direct arm of SM tendon as well as proximal division of the sMCL [11, 12, 17, 18] (Fig. 2a, step 2). This release procedure was performed approximately 2–5 cm from the joint line distally.

If the medial gap was still tight, tibial reduction osteotomy was performed until balancing of the joint gap medially to laterally as well as in extension and flexion was achieved. The tibial component was placed along the line of the lateral margin of the tibial surface. After the size and the location of the tibial component were decided, 4-mm vertical osteotomy along the medial aspect of the uncapped tibial surface was performed. According to the medial tightness of flexion and extension gaps, two different regions of tibial reduction osteotomy were selected for medial release. Medial tightness in flexion was released by medial tibial reduction osteotomy, which was parallel to the anteroposterior axis. Medial tightness in extension was released by posteromedial tibial reduction osteotomy, which inclined to the anteroposterior axis at an angle of 30° (Fig. 2b, step 3).

**Fig. 3:**
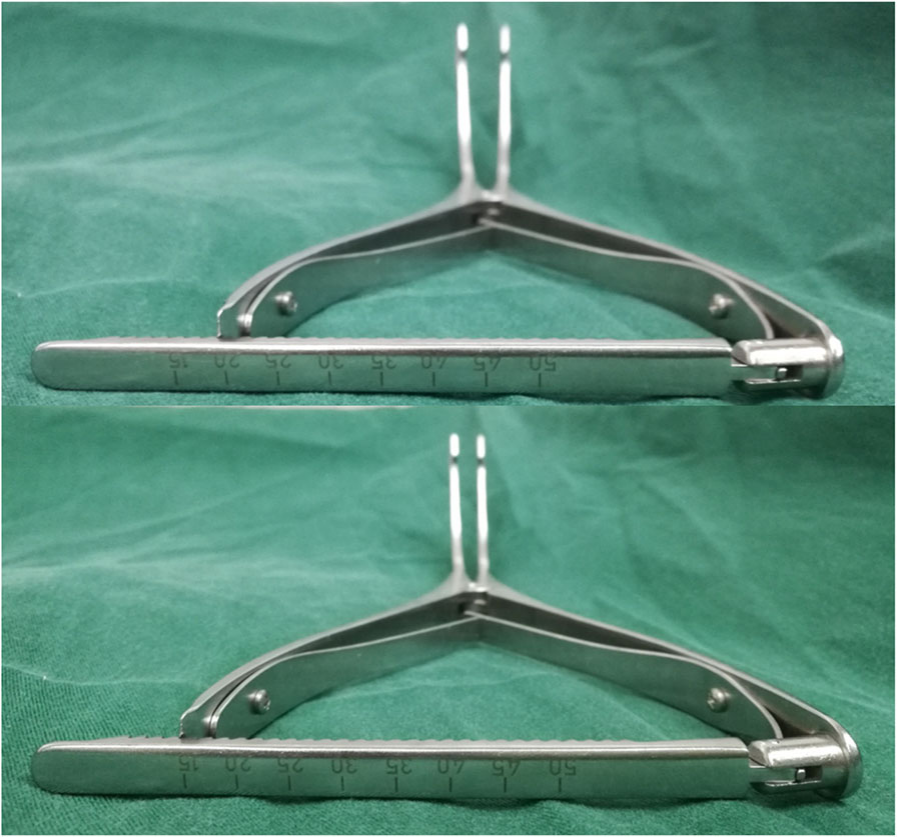
The spreader device with a torque meter provides a stepwise spreading by detents, and the step size is 1 mm

Measurements of the medial and lateral femorotibial joint gap width were performed in full extension and 90° of flexion step-by-step during surgery by a spreader device with a torque meter (Fig. 3). The spreader device was inserted into the joint to adequately distract the joint with the same extrusion force by the thumb and forefinger. The amount of tightness in the joint gap was recorded twice at each step of medial release, and the average value was calculated. Clinical outcomes were also evaluated by the range of motion, knee alignment parameter, and Knee Society Scores (KSSs). The postoperative standing full-length lower limb radiographs were taken, and the knee alignment was recorded.

Statistical analysis was performed with SPSS 16 software (SPSS Inc., Chicago, IL, USA). For numeric data, mean ± SD was calculated. Chi-square test was used to compare the ratio of gender and outliers of alignment among different groups. Overall comparison of the age, range of motion, KSS, and knee alignment among three groups was conducted by the analysis of variance (ANOVA). Statistical heterogeneity was considered to be present at *p* < 0.1; data were analyzed using ANOVA as no heterogeneity existed. Differences were considered as statistically significant with a *p* value of less than 0.05.

## Results

Patients’ demographics, imaging parameters, and relevant clinical scores are presented in Table 1. According to the step-by-step release of the medial structure of the knee, the patients were divided into three groups. In 276 of the 466 (59%) primary TKAs, symmetrical gaps could be achieved by the limited release of the capsule and dMCL within 2 cm from the joint line distally (group 1). In 152 cases (33%), release of adhesive structure was performed approximately 2–5 cm from the joint line distally and the distal attachment of sMCL was not released (group 2). In 38 out of the 466 (8%), the medial tightness necessitated a reduction osteotomy after the above procedures (group 3). There was no significant difference in age and gender ratio among the three groups (each *p* > 0.05, Table 1). There were significant differences in the preoperative knee alignment, preoperative range of motion, and preoperative KSS among the three groups (each *p* < 0.01, Table 1).

**Table 1:** Comparisons of demographics of patients in each group

	Group 1 (*n* = 276)	Group 2 (*n* = 152)	Group 3 (*n* = 38)	*p* value
	Mean ± SD	Mean ± SD	Mean ± SD	
Age (years)	63.8 ± 4.7	63.6 ± 5.7	63.9 ± 5.3	0.854
Gender (female/male)	169:107	94:58	28:10	0.326
Pre-range of motion	118.4 ± 7.0°	112.1 ± 10.6°	109.4 ± 8.9°	0.001
Post-range of motion	125.8 ± 6.6°	124.3 ± 5.9°	123.5 ± 6.4°	0.001
Pre-knee alignment (varus)	5.2 ± 2.1°	6.6 ± 2.2°	9.7 ± 2.6°	0.001
Post-knee alignment (varus)	0.6 ± 1.6°	0.8 ± 1.5°	1.2 ± 1.7°	0.222
Pre-KSS	63.7 ± 4.8	60.8 ± 5.9	56.8 ± 6.4	0.001
Post-KSS	89.7 ± 3.4	88.4 ± 4.1	87.5 ± 3.1	0.001
Outliers	10/276	6/152	3/38	0.457

*Post-* postoperative, *Pre-* preoperative, *KSS* Knee Society Score, *Outliers* deviation beyond 3° of varus or valgus mechanical axis, *Group 1* limited release of capsula and dMCL, *Group 2* release of adhesive medial structure (2–5 cm from the joint line distally), *Group 3* medial soft tissue release combined with tibial reduction osteotomy

The beneficial effects of medial soft tissue release and reduction osteotomy were evident on the analysis of joint gap kinematics. Medial gap width increased 1.6 ± 0.8 mm and 1.7 ± 0.6 mm at knee extension and flexion of 90°, respectively, following limited release of the capsula and dMCL (step 1). Medial gap width increased 1.0 ± 0.4 mm and 1.6 ± 0.5 mm at knee extension and flexion, respectively, following additional release of adhesive anterior-medial structure (step 2a). Likewise, the increase of the medial gap at knee extension and flexion was 1.7 ± 0.6 mm and 1.2 ± 0.5 mm, respectively, after release of adhesive posteromedial structure (step 2b). Regardless of the region of the reduction osteotomy performed, the medial gap significantly increased in both extension and flexion. The increase of the medial gap at knee extension and flexion of 90° was 1.1 ± 0.5 mm and 2.0 ± 0.8 mm, respectively, following medial reduction osteotomy (step 3a). Likewise, following posteromedial reduction osteotomy, the increase of medial gap at knee extension and flexion of 90° was 1.5 ± 0.8 mm and 0.8 ± 0.4 mm, respectively (step 3b). The final amount of medial structure release is shown in Table 2.

**Table 2:** Gap increases with each procedure in total knee arthroplasty

Surgical step	Extension		Flexion	
	Medial gap increasement (mean ± SD)	Lateral gap increasement (mean ± SD)	Medial gap increasement (mean ± SD)	Lateral gap increasement (mean ± SD)
Release of the dMCL (step 1)	1.6 ± 0.8	0.4 ± 0.2	1.7 ± 0.6	0.5 ± 0.3
Broad medial release (step 2)				
Anterior-medial release (2a)	1.0 ± 0.4 A	0.2 ± 0.2 E	1.6 ± 0.5 a	0.6 ± 0.2 e
Posteromedial release (2b)	1.7 ± 0.6 B	0.4 ± 0.3	1.2 ± 0.5 b	0.5 ± 0.3
Reduction osteotomy (step 3)				
Medial osteotomy (3a)	1.1 ± 0.5 C	0.2 ± 0.3 F	2.0 ± 0.8 c	0.6 ± 0.4 f
Posteromedial osteotomy (3b)	1.5 ± 0.8 D	0.3 ± 0.2	0.8 ± 0.4 d	0.4 ± 0.3

Each value is recorded as the increasement of gap after each surgical step: mean (mm) ± standard deviation (SD). *p* < 0.01 for A versus a, B versus b, C versus c, D versus d, E versus e, and F versus f using paired sample *t* test

*dMCL* deep medial collateral ligament

The range of motion improved from 118.4 + 7.0°, 112.1 + 10.6°, and 109.4 + 8.9° preoperatively to 125.8 + 6.6°, 124.3 + 5.9°, and 123.5 + 6.4° postoperatively at the last follow-up for groups 1, 2, and 3, respectively. The mean preoperative knee mechanical alignment was varus of 5.2 + 2.1°, 6.6 + 2.2°, and 9.7 + 2.6° and was corrected to 0.6 + 1.6°, 0.8 + 1.5°, and 1.2 + 1.7° postoperatively for groups 1, 2, and 3, respectively. The KSS improved from 63.7 + 4.8, 60.8 + 5.9, and 56.8 + 6.4 preoperatively to 89.7 + 3.4, 88.4 + 4.1, and 87.5 + 3.1 postoperatively for groups 1, 2, and 3, respectively. There was significant difference in postoperative KSS at the last follow-up among the three groups (*p* < 0.001, Table 1). No difference was noted when comparing postoperative knee alignment and ratio of outliers in the three groups (*p* = 0.222, *p* = 0.457, respectively, Table 1). No specific complications related to the present technique, such as nerve injury, hematoma formation, subsidence of the tibial component, or conversion to a constrained prosthesis due to over release, were observed.

## Discussion

**Fig. 4:**
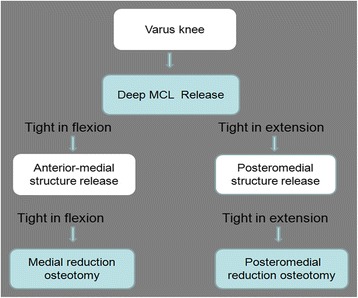
Algorithmic approach of the medial release based on the principle of selective functional release of the medial soft tissue structure and reduction osteotomy

We introduce a knee joint position similar to Patrick’s sign in favor of exposing the medial structure and established a step-by-step selective release procedure, which consisted of dMCL release, selective release of medial soft structure, and selective tibial reduction osteotomy (Fig. 4). A refined technique of selective medial release was performed using anterior-medial soft tissue release and medial tibial reduction osteotomy for tightness in flexion, while posteromedial structure release and posteromedial tibial reduction osteotomy for tightness in extension.

Subperiosteal release of posteromedial corner structures, such as the POL and the SM tendon fibers that merge into the posterior capsule, is often cumbersome in severe varus knee [17]. In the current study, a knee joint position similar to Patrick’s sign was used, which was effective for sufficient exposure of the medial structure and reduced the possibility of iatrogenic injury to the saphenous nerve.

Three important ligaments maintain primary medial knee stability: the sMCL, POL, and dMCL [11, 12]. The dMCL, whose attachment is on average about 6 mm distal to the tibial plateau, is often released as the first step of medial release during TKA in terms of efficacy and safety [6]. The POL is a primary restraint to internal rotation and is a secondary restraint to valgus translation and external rotation [19]. The POL and sMCL are described as having a complementary relationship in resisting internal rotation torque [20]. Significant increase of rotatory instability is seen on the release of the POL, and thus, retaining the POL has a possibility to improve the outcome after primary TKA [18].

The sMCL plays a consistent role in resisting both the isolated valgus moment and combined valgus and internal rotation moments. Since the proximal division of the sMCL and posterior part of the superficial medial ligament which is attached to the medial meniscus would be released during TKA, the function of the sMCL may be affected [12]. Because the integrity of the sMCL of the knee is crucial to the proper function and longevity of nonconstrained TKA, conservation of sMCL fibers at the tibial insertions, especially on the distal division (62.4 ± 5.5 mm distal to the joint line) is considered to be critical [11–13, 21]. In the current study, the tibial distal division of the sMCL was retained in all cases by using selective medial soft release (less than 5 cm distal to the joint line) combined with selective tibial reduction osteotomy.

It could be argued that the classic extensive medial release associated with iatrogenic injury to the pes anserine and saphenous nerve, instability, and abnormal knee kinematics may be unnecessary [10]. In addition, the concept of constitutionally varus alignment that restores pre-arthritic natural anatomical alignment is emerging in recent years [22–24]. Some studies have reported that kinematically aligned TKA without MCL release and restoration of the limb into patients’ natural alignment of slight varus has shown a favorable outcome [6]. Our previous research indicated that a mechanical axis within 3° of varus in the coronal plane remained a satisfactory target in TKA surgery [10]. Thus, mini-invasive release of the medial elements may allow for improved soft tissue balancing leading ultimately to improved functional outcome.

The reduction osteotomy increased component gap width and decreased the varus angle, which might reduce the risk of over release, as well as the amount of medial soft tissue release [25]. It could achieve higher KSS than the TKA series without reduction osteotomy potentially due to reduce medial soft tissue release [15]. There are some tips for tibial reduction osteotomy: firstly, it is important to keep the bone cutting level on the tibia as high as possible, which helps to prevent lateral cortex blowout and maintain the bone quality of the cutting surface [15]. Secondly, in the setting of reduction osteotomy, the tibial base plate is suggested to place along the bony margin of the lateral tibial plateau without downsizing. In the current study, 4-mm reduction osteotomy did not significantly decrease bone quality and a one size smaller tibial component was used in only 2 of 38 knees, where medial osteotomy was used.

Employment of a combined release technique seems to be more effective and minimally invasive than either medial soft structure release or tibial reduction osteotomy alone, since maximal tension exists at the initial phase of each release procedure. For example, 4-mm reduction osteotomy provides approximately 1.7° and 0.7° varus improvement in flexion and extension, respectively, while 8-mm reduction osteotomy only improves 2.8° and 0.9° in flexion and extension, respectively [15]. In addition, employment of a stepwise release technique could avoid unnecessary over release [7, 9].

In the current study, we provide a selective release technique according to extension-flexion gap balance. Release of the anterior-medial soft tissue and medial reduction osteotomy tends to increase the flexion gap more than the extension gap, whereas release of the posteromedial elements and posterior osteotomy tends to affect the extension gap more than the flexion gap. We believe that the results of this study provide valuable information for a TKA procedure to achieve better extension-flexion balancing. Various choices could be selected according to the extension-flexion balancing.

## Conclusion

The technique of the tibial reduction osteotomy combined with medial soft structure release using Patrick’s sign is effective, safe, and minimally invasive to obtain balanced mediolateral and extension-flexion gaps in primary TKA.
